# *Leishmania* spp. DNA in Sand Flies in a Transition Area of the Cerrado and Atlantic Forest biomes, Mato Grosso do Sul, Brazil

**DOI:** 10.1007/s13744-026-01424-4

**Published:** 2026-07-31

**Authors:** Iara Beatriz Andrade de Sousa, Marco Aurélio Louveira Areco, Gabrielle Lopes Noia, Anibal Salinas Junior, Kamily Fagundes Pussi, Jhoy Alves Leite, George Tadeu Nunes Diniz, Paulo Silva de Almeida, Manoel Sebastião da Costa Lima Junior, Herintha Coeto Neitzke-Abreu

**Affiliations:** 1 Postgraduate Program in Health Sciences, Universidade Federal da Grande Dourados, Mato Grosso Do Sul, Dourados, Brazil; 2 Faculty of Biological and Environmental Sciences, Universidade Federal da Grande Dourados, Dourados, Mato Grosso Do Sul Brazil; 3 Faculty of Health Sciences, Universidade Federal da Grande Dourados, Dourados, Mato Grosso Do Sul Brazil; 4 Municipal Entomology Laboratory, Zoonoses Control Center, Nova Andradina, Mato Grosso Do Sul Brazil; 5 Statistics and Geoprocessing Center, Instituto Aggeu Magalhães/Fiocruz, Recife, Pernambuco Brazil; 6 Regional Entomology Laboratory, Regional Health Center, Dourados, Mato Grosso Do Sul Brazil; 7 Laboratory of Immunopathology and Molecular Biology, Instituto Aggeu Magalhães/Fiocruz, Recife , Pernambuco Brazil

**Keywords:** Leishmaniasis; Phlebotominae; Disease vectors

## Abstract

Leishmaniases are infectious diseases whose vectors are female sand flies that depend on the blood of vertebrates for egg maturation. In this study, we investigated the sand fly species and DNA from *Leishmania* spp. This entomological/epidemiological study was conducted in a rural area in Nova Andradina, Mato Grosso do Sul, Brazil. From March 2019 to February 2020, five modified Falcão light traps were installed at night once a month. The collected insects were separated by sex into pools of 10 specimens. A sample was used for species identification via microscopic analysis, and another sample was used to detect the DNA of *Leishmania* in females via polymerase chain reaction (PCR). A total of 42,933 insects were collected, 77.6% (n = 33,313) of which were female. A total of 4,993 (11.6%) insects were identified, most were *Nyssomyia neivai* (n = 4,839; 96.9%). For PCR, 4,130 (9.6%) insects were analyzed, and four pools were positive for *Leishmania* spp. (with a natural infection rate of 0.1%). When the number of sand flies collected and the climatic variables were correlated, a significant difference was detected when precipitation > 60 mm (p = 0.0314) and relative humidity ≤ 60% (p = 0.0201). The analyzed area is inhabited by one of the main vectors of cutaneous leishmaniasis in Brazil, and we also found that some specimens were infected with *Leishmania* DNA, which might favor the occurrence of the disease in the region. This information is essential for understanding the dissemination of leishmaniasis and planning actions to prevent in the municipality.

**Supplementary Information:**

The online version contains supplementary material available at 10.1007/s13744-026-01424-4.

## Introduction

Leishmaniases are neglected infectious diseases with distinct clinical manifestations, include two forms—visceral leishmaniasis (VL) and cutaneous leishmaniasis (CL). With more than 1 million new cases per year, it is endemic to 98 countries, which are found mainly in Asia, Africa, and South America, revealing a high potential for public health problems in these regions (WHO 2025). According to cases reported by the Ministry of Health, both VL and CL in the state of Mato Grosso do Sul has stabilized, considering that the number of cases reported in the last eight years has remained close to 100. In 2024, 72 cases of CL and 105 of VL were diagnosed in humans, and 194 cases of CL and 159 of VL were diagnosed in 2023 (Brazil 2025a, b).

Mato Grosso do Sul has unique ecological and biogeographical characteristics, including transition zones between the Cerrado, Pantanal, and Atlantic Forest biomes, which favor a high diversity of phlebotomine sand flies and a wide vector distribution, with records in approximately 75% of the municipalities in the state, highlighting the need for continuous and regionalized entomological studies (Neitzke-Abreu et al. 2022). In the transmission of CL, several species of sand flies have been reported, the most frequent being *Migonemyia migonei* (França, 1920), *Nyssomyia neivai* (Pinto, 1926), *Nyssomyia whitmani* (Antunes & Coutinho, 1939), *Pintomyia fischeri* (Pinto, 1926), and *Pintomyia pessoai* (Coutinho and Barretto, 1940) (Neitzke-Abreu et al. 2014, 2022; Barrios et al. 2020).

Despite the information obtained from decades of studies on leishmaniasis, further information is required concerning the long persistence of the endemic disease in specific locations. The region of Nova Andradina is classified as sporadic transmission (below 2.4 cases/year), and has reported three cases of canine leishmaniasis in the period from 2019 to 2020. In December 2018, a case of CL was reported in the study area, which motivated the investigation of this topic (Sousa et al. 2023). Thus, the objective of this study was to describe the speciesof sand flies and examine the DNA of *Leishmania* spp. by conducting polymerase chain reaction (PCR) in sand flies in the rural area of the municipality of Nova Andradina, Mato Grosso do Sul.

## Materials and Methods

### Study Srea and Data Collection

**Fig. 1:**
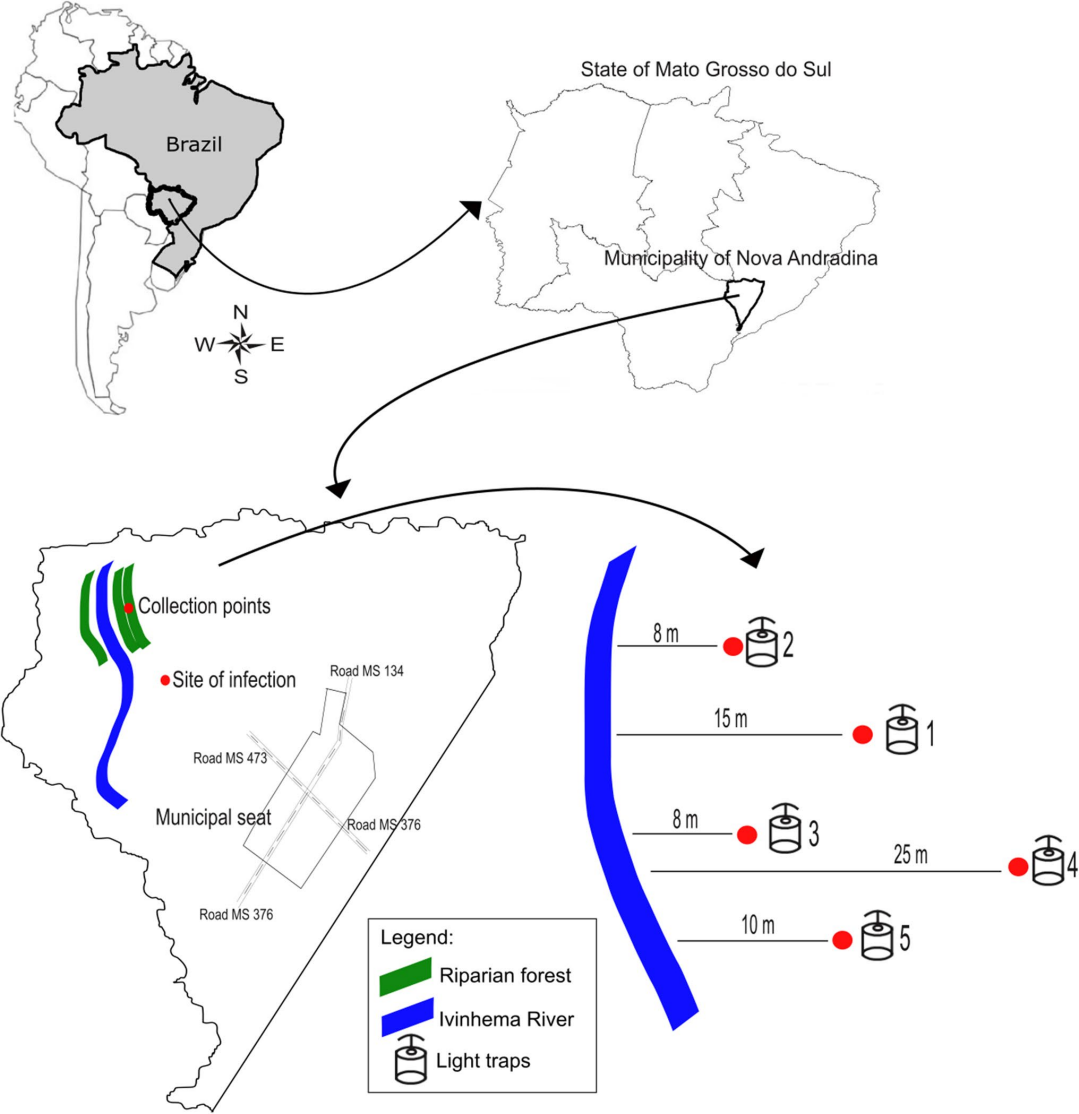
Geographical location of the study area and sand fly collection sites, at Fazenda Guarani, Nova Andradina, Mato Grosso do Sul, Brazil

Located in Mato Grosso do Sul, Brazil, the municipality of Nova Andradina (22° 14′ 00″ S and 53° 20′ 35″ W) covers an area of 4,768.118 km^2^ and has 48,563 inhabitants (IBGE 2022) (Fig. 1). The municipality belongs to the Paraná River Basin and is located in the Cerrado and Atlantic Forest biomes.

The samples were systematically collected in fragments of riparian forest on the bank of the Ivinhema River, which is located on a rural property (Fazenda Guarani). The samples were collected between March 2019 and February 2020; however, November and December were excluded because the area was inaccessible due to heavy rainfall and soil conditions. Five light traps of the modified Falcão (Falcão 1981) light traps (WM, Campo Grande, Brazil) were installed at five collection sites, all within the riparian forest; these sites were the most likely to contract the infection (night fishing) (Fig. 1), as mentioned by patient treated with CL. The pontis were: **a.** The probable site of infection (indicated by the patient in the case report) was about 4 m from the riverbank, with the soil beaten with red earth and stones; **b.** The trap installed at collection site 1 was about 15 m from the river bank on beaten red earth; **c.** The trap installed at collection site 2 was about 8 m from the riverbank, and the soil was beaten with red earth, with the presence of stones; the closest point to the place was indicated as the probable site of infection; **d.** The trap installed at collection site 3 was about 8 m from the riverbank; red earth soil with less compaction; **e.** The trap installed at collection site 4 was about 10 m from the riverbank, red earth soil with minor compaction, and soil covered by a layer of dry leaves; **f.** The trap installed at collection site 5 was about 25 m from the riverbank; red earth soil with minor compaction and soil covered by a layer of dry leaves. All collection sites had environmental characteristics favorable to sand flies ocorrence (the presence of human and animal circulation trails and the presence of organic matter) and proximity to a place used for fishing, with distances of about 15 m and 80 cm from the ground (Supplementary Fig. S1). The traps were placed from 4 p.m. to 8 a.m. for one day each month. At each collection site, the traps were laid for 160 h, totaling 800 h of collection.

Data on climatic variables (temperature and relative humidity) for the study period were collected from the forest fragments at the time the traps were installed. The accumulated monthly precipitation data were obtained from Cooperativa Agrícola Sul Matogrossense (COPASUL).

### Processing of Sand Flies

After collection, the sand flies were separated by sex, refrigerated in microtubes containing 80% alcohol at the Entomology Laboratory of the Zoonoses Control Center of Nova Andradina, and sent for further processing to the Entomology Laboratory of the Regional Health Center of Dourados. Some of the sand flies collected were used for species identification; some female sand flies were stored at –20 °C, with pools of 10 specimens from the same collection site and date of collection, for detecting the DNA of *Leishmania* spp. via PCR. The other insects were used to count the total number of sand flies collected.

Sand fly species were identified at the Entomology Laboratory of the Regional Health Center of Dourados. To identify the species of sand flies, the Berlese preparation technique in males and females was used (Forattini 1973). Morphological identification was performed following the method proposed by Galati (2018), and the abbreviation of phlebotomine genera followed the classification proposed by Marcondes (2007).

Polymerase chain reaction of the female sand flies was performed at the Health Sciences Research Laboratory at Universidade Federal da Grande Dourados (UFGD). Pools with female sand flies were dried in a dry block at 80 °C for 10 min to obtain DNA. Next, they were extracted following the method described by Loxdale and Lushai (1998), with modifications. They were macerated with a sterile spatula with 300 μL of Chelex™ Molecular Biology Grade Resin (Bio-Rad Laboratories, São Paulo, Brazil) at 5%. The contents were mixed with a vortex tube stirrer and heated in a water bath at 80 °C for 30 min. Subsequently, the mixture was centrifuged, and the supernatant was removed, transferred to another sterile microtube, and stored at –20 °C for at least 12 h for PCR. Positive extraction controls (pool of male sand flies with *Leishmania* spp. promastigotes) and negative extraction controls (pool of male sand flies without *Leishmania* spp. promastigotes) were used.

To detect *Leishmania* spp., multiplex PCR was performed with two pairs of primers, 13A/13B (Rodgers et al 1990), aimed at amplifying a 120-bp fragment present in different species of *Leishmania,* and 5Llcac/3Llcac (Lins et al. 2002) for reaction control and DNA integrity, aimed at amplifying a 220-bp fragment of the region of the IVS6 gene of cacophony in insects, following a previously established protocol (Pussi et al. 2023). Positive PCR control (*Leishmania* spp. DNA) and PCR control negative (water) were used. The amplified products were subjected to 2% agarose gel electrophoresis and stained with ethidium bromide. Bands were detected on a transilluminator.

### Statistical Analysis

Owing to the high number of sand flies collected, a sample calculation was performed (Supplementary Fig. S2a) to identify the species in which an infinite population was considered, with an approximated expected frequency in an initial screening of specimens and a prevalence of 97.3% of *Ny. neivai* (Oliveira et al. 2011), a sampling error of 5%, and a confidence level of 95%, resulting in a minimum sample of 4,037. To calculate the detection rate of *Leishmania* spp., the formula for sample proportion calculation was used (Supplementary Fig. S2b), considering a population of 33,313 female sand flies, the expected prevalenceof 0.22% of *Leishmania* DNA in *Ny. neivai* (Oliveira et al. 2011), the acceptable sampling error of 0.14%, and a 95% confidence interval, with a minimum sample size of 3,810.

The data were previously evaluated via a parametric or non-parametric approach. Thus, the variables were tested for their normality distribution using the Shapiro–Wilk test. When the data followed a normal distribution, Student’s t-tests were performed to determine the existence of differences between means. However, when the data did not follow a normal distribution, the Mann–Whitney test was performed to evaluate the differences between the medians.

The correlations between the climatic variables (temperature, humidity, and precipitation) and the number of sand flies collected (total, males and females) monthly were determined using Spearman correlation coefficients (r). The Mann–Whitney test was used to compare sand flies abundance among groups defined by climatic variables (temperature ≤ 27 °C and > 27 °C, relative humidity ≤ 60% and > 60%, and precipitation ≤ 60 mm and > 60 mm). The level of significance was set at 5% (p < 0.05) with a 95% confidence interval (CI 95%). All statistical analyses were performed in R version 3.5.1 (R Core Team 2018).

## Results

A total of 42,933 insects were collected (hourly average of 53.7), most of which were female (n = 33,313; 77.6%), with a male/female ratio of 0.3:1. A comparison of the medians of the sand flies captured throughout the study (Mann–Whitney test) revealed that the median number of females (764) was greater than that of males (167.5) (U = 25, Z = –1.890, p = 0.063).

**Fig. 2:**
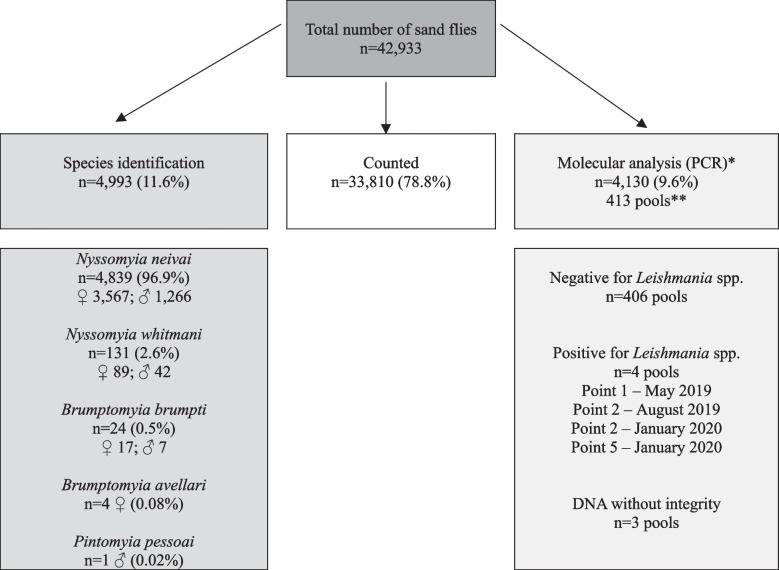
Collection flowchart, species identification, and molecular analysis; Nova Andradina, Mato Grosso do Sul, 2019–2020 Legend: *PCR: polymerase chain reaction; **In each pool, 10 female samples were collected on the same day from the same collection site.

Among the identified samples, *Ny. neivai* was the predominant species (n = 4,839; 96.9%). Four other species were found, including *Ny. whitmani* (n = 131; 2.6%), *Brumptomyia brumpti* (Larrousse, 1920) (n = 24; 0.5%), *Brumptomyia avellari* (Costa Lima, 1932) (n = 4; 0.08%), and *Pi. pessoai* (n = 1; 0.02%) (Fig. 2).

**Fig. 3:**
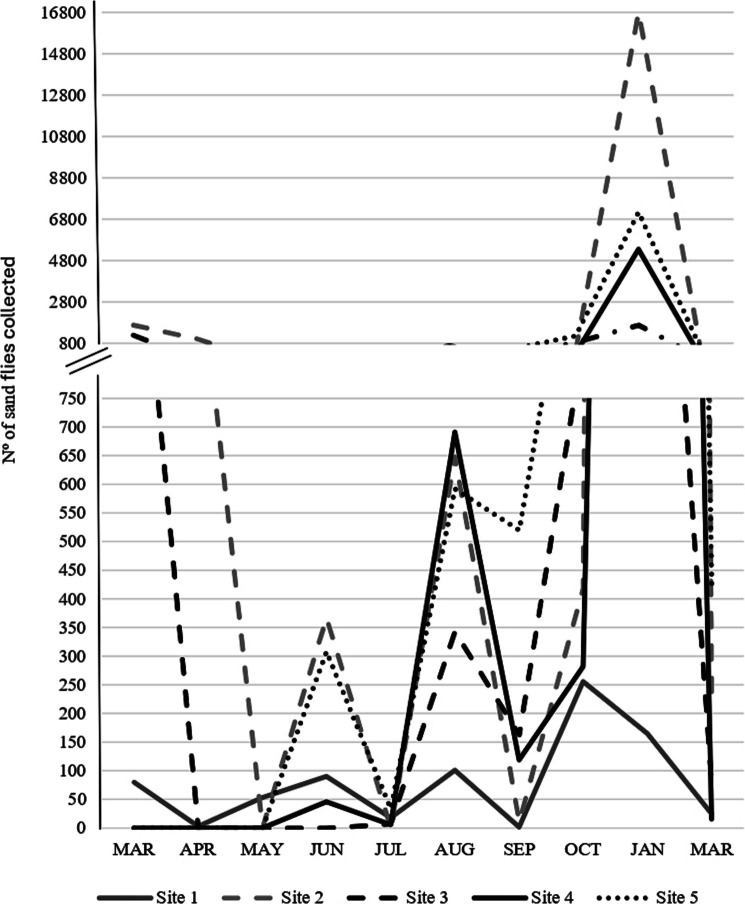
Number of sand flies by site for each month, Nova Andradina, Mato Grosso do Sul, Brazil, 2019–2020

The number of sand flies collected per month at different sites is presented in Fig. 3. Collection site 1 (n = 793; 1.8%) had the lowest percentage, in contrast to collection site 2, which had the highest number of specimens collected (n = 21,154; 49.3%). The number of specimens collected in collection sites 3, 4, and 5 was 4,299 (10%), 6,509 (15.2%), and 10,178 (23.7%), respectively. For population density, more than 1,000 samples/month were captured in 50% of the months, with a peak in January 2020, when about 31,137 (72.5%) sand flies were collected. In May 2019 and July 2019, the lowest number of sand flies were collected (n = 53; 0.12% and n = 66; 0.15%, respectively).

Of the female samples that were selected for PCR analysis, only four of the 410 pools were positive for *Leishmania* spp. (representing a DNA detection rate of 0.1%); however, two positive pools were from collections performed in January 2020, which was the month in which a relatively high number of insects were collected (Fig. 2).

**Fig. 4:**
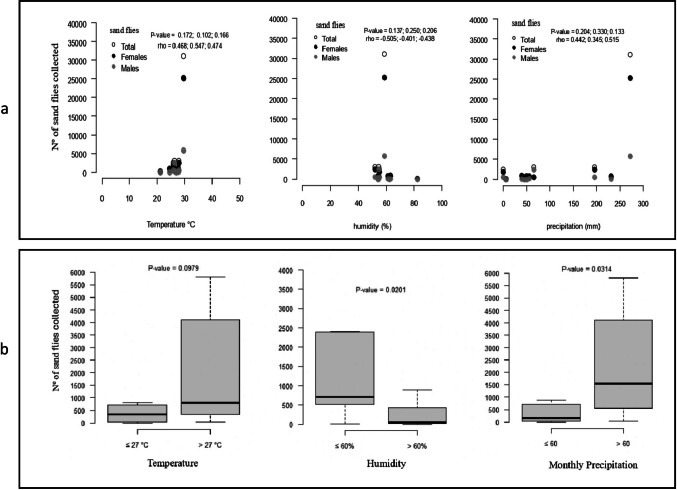
a. Correlation graphs between the number of sand flies (total, female, and male) and climatic variables (temperature, monthly precipitation index, and relative humidity) by Spearman. **b.** Number of total sand flies and climatic variables (temperature ≤ 27 °C and > 27 °C, relative humidity ≤ 60% and > 60%, and precipitation ≤ 60 mm and > 60 mm) by Mann–Whitney. Nova Andradina, Mato Grosso do Sul, Brazil, 2019–2020

During the 10 months of collection, there were differences in the reference values of temperature, air humidity, and precipitation. The mean temperature was 21.9 ± 10.5 °C, the humidity was 66.4 ± 17.6%, and the precipitation was 119 ± 108.6 mm. When the number of sand flies collected was correlated with temperature (r = 0.468; p = 0.172), humidity (r = –0.505; p = 0.137) and rainfall (r = 0.442; p = 0.204) no significant association was observed by Spearman (Fig. 4a). When stratified by sex, males and females also did not show a statistically significant correlation between abundance and climatic variables. When comparing sand flies abundance across climatic groups, higher abundance was observed during months with precipitation exceeding 60 mm (p = 0.0314) and periods with relative humidity of 60% or less (p = 0.0201) by Mann–Whitney. Regarding temperature, although higher abundance was observed in months with temperatures above 27 °C, this difference was not significant (p = 0.0979) (Fig. 4b).

## Discussion

Before sand fly collection commenced, there was a record of a CL case involving a worker at the farm close to the sampling points (Sousa et al. 2023). Based on this information, the sites of collection were selected in the vicinity where the worker reported performing leisure activities (fishing) on the banks of the Ivinhema River in a period conducive to the presence of the vector (dark hours, without the use of repellent or appropriate clothing). The Ivinhema River is widely used for fishing and may indirectly influence the incidence of cases of *Leishmania* spp., considering that people visit the river, especially at night, and many people are unaware of preventive measures to control the transmission of CL.

Interestingly, the number of samples collected was high (n = 42,933), with an hourly average of 53.7 (these data correspond to the sum of the traps placed at the sites of collection), which is much greater than that reported in most fauna studies within and outside the state of Mato Grosso do Sul (Barrios et al. 2019; Oliveira et al. 2011; Silva et al. 2022). There were more females (33,313) than males (9,620), with a male-to-female ratio of 0.3:1. This was similar to the finding reported by Neitzke et al. (2008), who worked in an endemic area of Paraná and collected a large number of sand flies, with a male-to-female ratio of 0.5; Silva et al. (2022) found a male/female ratio of 3. Some researchers have found a predominance of males and reported that this occurs due to natural behavior, in which males accompany females to ensure fertilization and are more attracted to light traps (type CDC) (Barrios et al. 2019; Silva et al. 2022). Costa et al. (2019) reported that females outnumber males in forest areas, and the relationship is reversed in residential areas. The high number of females recorded in this study may be related to the behavior and habits of searching for blood for egg maturation, taking advantage of the presence of vertebrate hosts in these environments.

The sand fly species found in the study area of Nova Andradina showed little diversity, with a predominance of *Ny. neivai*, accounting for 96.9% (n = 4,839) of the total number of samples identified, this fact was also reported by Teodoro et al. (2011), who found a predominance of *Ny. neivai* in a locality close to the Ivaí River (Paraná), where people visited for fishing and leisure. The findings of this study agree with the entomological research conducted by Eckert and Souza (2010) and Almeida et al. (2013) in Rio Grande do Sul and Mato Grosso do Sul, respectively, where there is a high frequency of *Ny. neivai* specimens. A study conducted in an urban area of Nova Andradina by Leite (2015) revealed a low number of *Ny. neivai* in the region, but owing to its adaptability to the availability of hosts and environmental changes with adjustments in its feeding habits, its population could change over time. This species is involved in transmitting the causative agents of CL in several endemic areas in Brazil (Andrade Filho et al. 2007). Therefore, owing to its epidemiological importance and high population density, it may be involved in the CL transmission cycle in the study area, considering that the high density of vectors increases the possibility of transmission in these locations (Neitzke-Abreu et al. 2014; Leite 2015).

*Nyssomyia whitmani* was the second most frequent vector, accounting for 2.6% (n = 131) of the analyzed sample. Despite the low frequency, its vectorial capacity has been determined in other regions of Brazil, and it has been observed that in areas where CL occurs, this species is in the process of domiciliation. Its spatial distribution is directly related to deforestation rates and inversely related to socioeconomic development. It is present in the state of Mato Grosso do Sul in the cities of Corguinho, Bodoquena, Bela Vista, Antônio João, and Dourados, both in forest regions and in caves (Galati et al. 1996; Nascimento et al. 2007; Santos 2010).

This study revealed that the other species were in a significantly reduced form, which may have occurred because *Ny. neivai* is exerting selective pressure on the local species that were identified in relatively small quantities. Almeida et al. (2013) also reported a low population of these species in forest regions, suggesting that this low number is directly associated with the effect of anthropization and the behavior of *Leishmania* vectors.

A large number of samples were collected at collection site 2, which was located on the banks of the Ivinhema River in an area whose treetops were relatively high and had clearings used for fishing activity (human modification). This resulted in more effective performance of light traps by allowing greater visibility and exposure. During the study period, 21,154 sand flies were captured at this collection site, representing 49.3% of the total. This occurred mainly due to environmental conditions, which were conducive to the reproduction and passage of different species of vertebrate animals that can serve as food sources for sand flies (Dias et al. 2007; Capucci et al 2023).

The percentage of *Leishmania* spp. DNA detected in this study (0.1%) is consistent with rates reported in the literature. A study in Corumbá (Mato Grosso do Sul) detected rates of natural infection by *L. infantum* of 1.5% and 0.7% in sand flies of the species *Lutzomyia cruzi* and *Lutzomyia forattinii*, respectively (Pita-Pereira et al. 2008). A study conducted in Paraná reported that 97.3% of the sand flies captured were from *Ny. neivai*, with a minimum infection rate of 0.22% for *Leishmania* spp. (Oliveira et al. 2011). In Florianópolis (Santa Catarina), natural infection rates of 1.6% were found for *Ny. neivai* (Dias et al. 2013). The PCR findings of this study confirmed the epidemiological data collected by Almeida et al. (2015) and Killick-Kendrick (1990), who regarded Mato Grosso do Sul as an endemic region for leishmaniasis with the presence of clinical cases diagnosed throughout the territory.

Climatic factors significantly influence the population density, eating habits, and movement of sand flies (Rebêlo et al. 2019). During the 10 months of collection, differences were recorded in the reference values of rainfall and air humidity. This oscillation in climatic variables is characteristic of the biome in the Mato Grosso do Sul region (Cerrado, Atlantic Forest, and Pantanal) (Marcuzzo et al. 2012), which favors the high diversity of sand flies and highlights the importance of studying and understanding the sand flies present in these biomes.

The findings of this study, despite the population density of sand flies and the correlation with climatic factors, agree with the conclusions of Rebêlo et al. (2019), who stated that to understand the manifestation and proliferation of *Leishmania* vectors, it is relevant to study the climatic and environmental components of Mato Grosso do Sul because of the non-homogeneity of climatic variants and the differentiation of urbanization between mesoregions.

In this study, no Spearman correlation was found between the density of sand flies and the temperature and relative humidity of the air, and rainfall, possibly due to their low variation during the study period, along with the fact that the duration of sample collection (10 months) may not have been sufficient to analyze such variables. On the other hand, the Mann–Whitney test revealed a higher abundance of sand flies during months with precipitation exceeding 60 mm and periods with relative humidity of 60% or less, suggesting that certain climatic conditions may favor the occurrence of these insects, even in the absence of a linear association between the variables. The greater abundance observed during the rainiest months aligns with studies conducted in Mato Grosso do Sul, which also reported an increase in phlebotomine populations during the rainy season. This behavior may be explained by the increased availability of breeding sites and organic matter, which favors the development of immature stages. Regarding relative humidity, although some literature describes higher phlebotomine densities in more humid environments, the results suggest that local environmental factors may exert a greater influence on the abundance of these insects than atmospheric humidity alone (Barrios et al. 2019).

With the increase in the population's interest in rural leisure in the region, the data from this study can contribute to the dissemination of knowledge about the epidemiology of leishmaniasis in the region, with the wide dissemination of protection measures and guidelines aimed at those who visit the locality. In this study, all collection sites were inside the riparian forest because there was less human interference with the environmental dynamics, which guaranteed greater diversity of species. The multiplicity of *Leishmania* vector species in Mato Grosso do Sul reflects the role of the Midwest region as a space for disseminating this disease in Brazil.

## Conclusions

The sand fly species in the analyzed area of Nova Andradina presented high population density and low diversity, with the identification of only five species. The predominant species was *Ny. neivai* at all collection sites. The detection of DNA from *Leishmania* spp. in some samples suggested the involvement of this species in the CL transmission cycle among people who use the studied area for leisure activities. Given the lack of knowledge about the sand fly species and the percentage of *Leishmania* spp. DNA in these insects in the municipality of Nova Andradina, this study contributes to the knowledge of the epidemiology of leishmaniasis in Mato Grosso do Sul. This information can improve our understanding of the dissemination of leishmaniasis and help us understand and plan prevention measures in the municipality.

## Supplementary Information

Below is the link to the electronic supplementary material.

Supplementary file1 **Fig. S1. **Sites of sand fly collection at Fazenda Guarani, Nova Andradina, Mato Grosso do Sul, Brazil (TIF 2254 KB)

Supplementary file2 **Fig. S2. **Formulae used for statistical analysis. **a.** The formula for sample calculation of proportions for an infinite population. **b.** The formula for the sample calculation of proportions (JPG 12 KB)

## Abbreviations

CL: Cutaneous leishmaniasis
COPASUL: Cooperativa Agrícola Sul Matogrossense
MS: Mato Grosso do Sul
PCR: Polymerase Chain Reaction
UFGD: Universidade Federal da Grande Dourados
VL: Visceral leishmaniasis
