# Supplementary figures and images for: Leishmania spp. DNA in Sand Flies in a Transition Area of the Cerrado and Atlantic Forest biomes, Mato Grosso do Sul, Brazil
Source: Neotrop Entomol. 2026 Jul 31;55(1):73. doi: 10.1007/s13744-026-01424-4 (PMC13427917; doi:10.1007/s13744-026-01424-4)

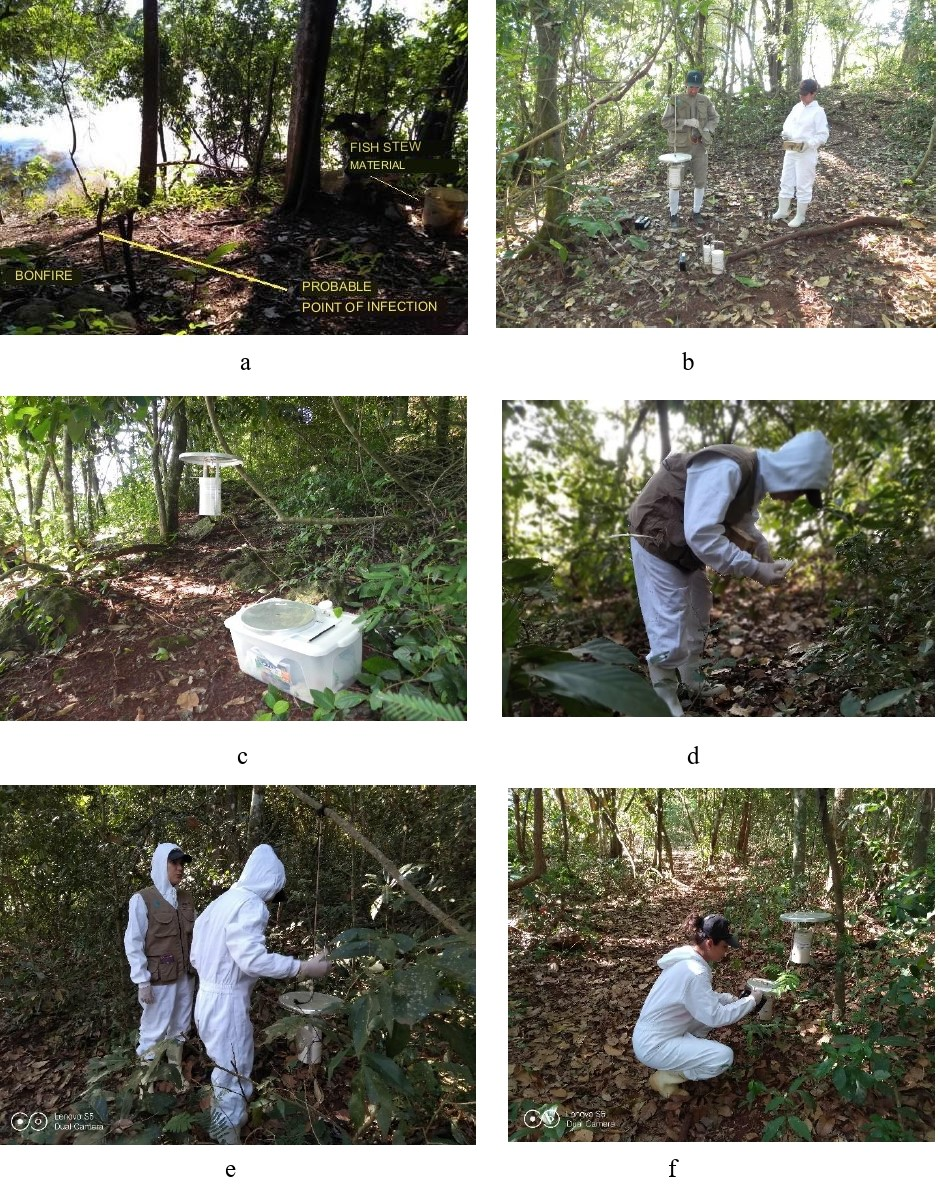

Supplement: Supplementary file 1 — Supplementary file1 Fig. S1. Sites of sand fly collection at Fazenda Guarani, Nova Andradina, Mato Grosso do Sul, Brazil (TIF 2254 KB) [file 13744_2026_1424_MOESM1_ESM.tif]

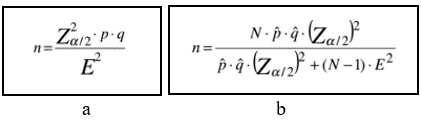

Supplement: Supplementary file 2 — Supplementary file2 Fig. S2. Formulae used for statistical analysis. a. The formula for sample calculation of proportions for an infinite population. b. The formula for the sample calculation of proportions (JPG 12 KB) [file 13744_2026_1424_MOESM2_ESM.jpg]
